# Education-related variation in coronary procedure rates and the contribution of private health care in Australia: a prospective cohort study

**DOI:** 10.1186/s12939-020-01235-y

**Published:** 2020-08-14

**Authors:** Veronica Hughes, Ellie Paige, Jennifer Welsh, Grace Joshy, Emily Banks, Rosemary J. Korda

**Affiliations:** 1 National Centre for Epidemiology and Population Health, Research School of Population Health, Australian National University, Canberra, ACT Australia; 2 Sax Institute, Sydney, NSW Australia

**Keywords:** Socioeconomic inequalities, Socioeconomic position, Education, Disadvantage, Coronary procedures, Myocardial infarction, Angina, Angiography, Revascularisation

## Abstract

**Background:**

Contemporary Australian evidence on socioeconomic variation in secondary cardiovascular disease (CVD) care, a possible contributor to inequalities in cardiovascular disease outcomes, is lacking. This study examined the relationship between education, an individual-level indicator of socioeconomic position, and receipt of angiography and revascularisation procedures following incident hospitalisation for acute myocardial infarction (AMI) or angina, and the role of private care in this relationship.

**Methods:**

Participants aged ≥45 from the New South Wales population-based 45 and Up Study with no history of prior ischaemic heart disease hospitalised for AMI or angina were followed for receipt of angiography or revascularisation within 30 days of hospital admission, ascertained through linked hospital records. Education attainment, measured on baseline survey, was categorised as low (no school certificate/qualifications), intermediate (school certificate/trade/apprenticeship/diploma) and high (university degree). Cox regression estimated the association (hazard ratios [HRs]) between education and coronary procedure receipt, adjusting for demographic and health-related factors, and testing for linear trend. Private health insurance was investigated as a mediating variable.

**Results:**

Among 4454 patients with AMI, 68.3% received angiography within 30 days of admission (crude rate: 25.8/person-year) and 48.8% received revascularisation (rate: 11.7/person-year); corresponding figures among 4348 angina patients were 59.7% (rate: 17.4/person-year) and 30.8% (rate: 5.3/person-year). Procedure rates decreased with decreasing levels of education. Comparing low to high education, angiography rates were 29% lower among AMI patients (adjusted HR = 0.71, 95% CI: 0.56–0.90) and 40% lower among angina patients (0.60, 0.47–0.76). Patterns were similar for revascularisation among those with angina (0.78, 0.61–0.99) but not AMI (0.93, 0.69–1.25). After adjustment for private health insurance status, the HRs were attenuated and there was little evidence of an association between education and angiography among those admitted for AMI.

**Conclusions:**

There is a socioeconomic gradient in coronary procedures with the most disadvantaged patients being less likely to receive angiography following hospital admission for AMI or angina, and revascularisation procedures for angina. Unequal access to private health care contributes to these differences. The extent to which the remaining variation is clinically appropriate, or whether angiography is being underused among people with low socioeconomic position or overused among those with higher socioeconomic position, is unclear.

## Background

Ischaemic heart disease (IHD) is the leading cause of mortality and morbidity globally and in Australia [1, 2] and the burden of IHD is higher in more socioeconomically disadvantaged groups [3–5]. The prevalence of biomedical and behavioural risk factors, and high absolute cardiovascular disease (CVD) risk is disproportionately high in people of lower socioeconomic position [6], as is incidence of the disease [7]. People of lower socioeconomic position are also more likely to have a major secondary cardiovascular disease (CVD) event [7] and are more likely to die from the disease [8]. Understanding the reasons for these differences is crucial to improving outcomes for more disadvantaged groups and lowering the overall burden of the disease.

Socioeconomic gradients in CVD events and mortality are likely to reflect, at least in part, differences in uptake and continuation with secondary prevention strategies, including coronary procedures [9, 10]. Angiography and revascularisation procedures, specifically percutaneous coronary intervention (PCI) and coronary artery bypass grafting (CABG), are recommended for the management of IHD, including acute myocardial infarction (AMI) and unstable angina [11]. Angiography is performed as a diagnostic procedure and, where clinically indicated, revascularisation procedures are used to improve blood flow to the heart [11, 12].

International and Australian studies have shown lower rates of coronary procedures among more disadvantaged populations [10, 13–15]. Recent findings suggest that these socioeconomic disparities may be disappearing over time as the overall use of coronary procedures has increased [16–20]. However, these studies have relied on area-level measures to assess socioeconomic position [13, 15, 16, 21, 22], which generally underestimate variation compared to individual-level measures [10, 23, 24]. Furthermore, many studies use data from public hospitals only, also potentially underestimating variation within the population, and providing no insights into the role of private health care in explaining inequalities in care. Australia has a hybrid health care model in which care is provided under the public system but people can opt to pay for additional private insurance [25]. The public component of the health care system, Medicare, covers the cost of public hospital admissions and some other health costs. Optional private health insurance provides access to private hospitals and covers costs of other health services not covered by Medicare. Private health insurance can reduce wait times for procedures; median wait times for private patients are around half that of public patients [26]. Around 43% of the Australian population had private health insurance in 2006 [26]. In addition, a small proportion of the population have access to private health care covered under the Department of Veterans’ Affairs Scheme. Contemporary Australian data using individual-level socioeconomic position and procedure data from both public and private hospitals are needed to provide evidence to inform clinical practice and the equitable use of coronary procedures following IHD events.

The aim of this study was to quantify the relation of education, a measure of individual-level socioeconomic position, to receipt of coronary procedures among patients admitted to public and private hospitals for AMI or angina. We also examined whether private health insurance explained any observed associations.

## Methods

### Data sources

Data for this study came from the Sax Institute’s 45 and Up Study, a prospective cohort of more than 267,000 men and women aged 45 years and over from New South Wales (NSW), Australia. Details on the study protocol have been published previously [27]. Briefly, participants were randomly sampled from the Department of Human Services database (formerly Medicare Australia enrolment database), which provides near-complete coverage of the population. People 80 years or older and residents of rural and remote areas were oversampled. Participants joined the Study by completing a baseline questionnaire (between January 2006 and December 2009) and giving signed consent for follow-up and linkage of their information to routine health databases. About 18% of those invited participated, with the final sample equivalent to 11% of the NSW population aged 45 years and over.

Baseline questionnaire data were linked to the NSW Admitted Patient Data Collection (APDC) which provides hospitalisation data – including information relating to admission dates, and diagnosis and procedure codes – for all patients admitted to NSW public and private hospitals. Data from the NSW APDC were available from 1 July 2001 until 30 June 2016. The data were also linked to the NSW Registry of Births, Deaths and Marriages and the National Death Index which provides date of death, used in this study for censoring. Data linkage was done probalistically by the Centre for Health Record Linkage [28].

### Study population

Participants were included in this study if they were admitted to a NSW public or private hospital with a primary diagnosis of AMI or angina after completing the baseline questionnaire and before 30 June 2016. AMI and angina were identified using International Statistical Classification of Diseases and Related Health Problems, Tenth Revision, Australian Modification (ICD-10-AM) codes I20 and I21, respectively. In order to better capture angina cases, participants hospitalised with a primary diagnosis of chronic ischaemic heart disease (IHD; ICD-10-AM code: I25) and a secondary diagnosis of angina were also included. Participants were stratified into separate AMI and angina samples. Participants who were hospitalised with both AMI and angina during the study period were categorised based on their first hospitalisation, with those recorded as having both AMI and angina in the same admission included in the AMI sample as this was considered to be the more clinically significant event.

**Fig. 1:**
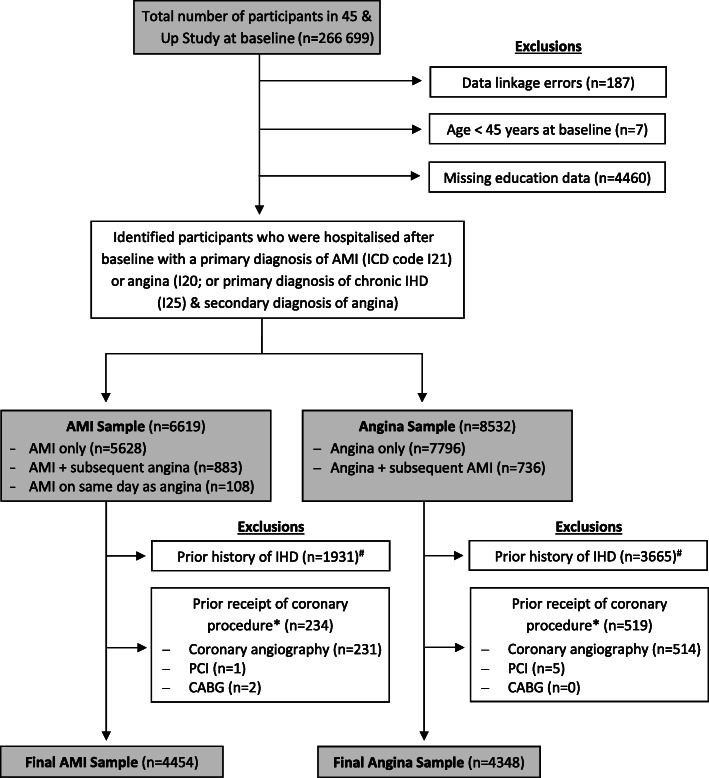
Sample selection. Note: AMI= acute myocardial infarction; CABG = coronary artery bypass grafting; IHD = ischaemic heart disease; PCI = percutaneous coronary intervention. ^#^Prior history of IHD refers to either self-reported heart disease from the baseline questionnaire or hospitalisation with any IHD diagnosis before baseline (linked data available from 1 July 2001). *Prior receipt refers to documented receipt of a coronary procedure in linked hospitalisation records (data available from 1 July 2001) before baseline

Participants were excluded if they had data linkage errors, were less than 45 years of age at baseline or were missing data on education, the main exposure. Participants were also excluded if they had a history of prior IHD, based on self-reported heart disease on the baseline questionnaire or hospitalisation with any IHD diagnosis (ICD-10-AM code: I20–25) or coronary procedure (angiography, CABG or PCI; procedure codes in Additional file 1) in the 6 years prior to baseline (see Fig. 1).

### Outcomes: receipt of coronary angiography and revascularisation procedures

The two outcomes were receipt of coronary angiography and receipt of coronary revascularisation, defined as either PCI or CABG, within 30 days of the index hospital admission. Outcomes were identified using Australian Classification of Health Interventions (ACHI, 8th edition) codes in any of the 50 procedure code fields in linked hospitalisation records (Additional file 1).

### Main exposure: education level

Socioeconomic position was measured using education attainment. Education was used because it is an individual rather than area-level measure, it is relatively stable over time [24, 29] and has little missing data (1.7%). Highest educational qualification attained was self-reported on the baseline questionnaire and grouped into three categories: low (no school certificate or other qualifications), intermediate (school or leaving certificate, trade, apprenticeship, diploma or other certificate) and high (university degree or higher) education.

### Other variables of interest

Variables included in models included sex (female, male), remoteness (major cities, inner regional or outer regional/remote/very remote; derived from the participant’s postcode of residence using the Accessibility/Remoteness Index of Australia [ARIA; 2011]), country of birth (Australia/New Zealand or other), body mass index (BMI, in kg/m^2^; underweight [BMI:15– < 18.5], normal weight [18.5–24.9], overweight [25–29.9] or obese [30–50] [30]), physical functioning (categorised based on the Medical Outcomes Study Physical Functioning Subscale [PF-10] as no or minor [PF-10 score: 90–100], moderate [score 60–89.9] or severe [score < 60] limitations [31, 32]) comorbidities (none, one, two or more, measured using the Charlson index and derived from hospitalisation records [33]). Private health insurance was self-reported on the baseline questionnaire, and was categorised as either yes (private hospital insurance or a Department of Veterans’ Affairs card) or no. All variables, except the Charlson Index, were recorded at the time of the baseline survey (2006–2009). The Charlson index was calculated using hospitalisation data and included comorbidities at time of index hospitalisation.

### Statistical analysis

Our analysis was performed in three stages. First, crude coronary procedure rates in relation to education level were calculated. Second, we used Cox proportional hazards regression models to estimate hazard ratios (HRs) and 95% confidence intervals (95% CI) for the receipt of coronary angiography and revascularisation (analysed separately) in relation to education level, with age at hospital admission as the underlying time variable. Participants were followed from the date of the first hospital admission for AMI or angina until they either experienced the outcome (received an angiogram or a revascularisation procedure), died due to any cause, or reached the end of the study (30 June 2016), whichever came first, for a maximum period of 30 days. Angina patients who were subsequently hospitalised for an AMI during follow-up (*n* = 118, 2.7%) were also censored on the date of the AMI admission to ensure that any coronary procedures received related to the initial angina event, not the subsequent AMI. In the third stage, we performed a subgroup analysis for the receipt of revascularisation among patients who had received angiography to assess whether any observed variation in revascularisation could be due to differences in angiography.

For each outcome, four models were used. Model 1 included education and sex, Model 2 additionally included remoteness and country of birth, and Model 3 additionally included BMI, physical functioning and number of comorbidities. Model 4 further included private health insurance. High education was used as the reference category and tests for linear trend in education were performed by including education as a continuous variable. Missing data for each of the covariates were included as separate categories in the analyses. For all models, the proportional hazards assumption was verified based on Schoenfeld residuals using a significance level of 0.01. Stratified Cox models were used where covariates did not satisfy the proportional hazards assumption. Post-hoc, where no association was seen between education and coronary procedures (Model 3), we assessed whether this was due to inequalities decreasing over time by stratifying results by receipt of procedure in two time periods (2006–10 and 2011–16).

Data were accessed through the Secure Unified Research Environment [34] and all analyses were performed using Stata version 14.1 [35].

## Results

After applying study exclusions (Fig. 1), the AMI and angina samples consisted of 4454 and 4348 participants, respectively. Sample characteristics are in Table 1 (AMI patients) and Table 2 (angina patents). Mean age was 73.2 years (standard deviation [SD] = 11.6) for the AMI patients and 70.3 years (SD = 10.3) for the angina patients. In the AMI sample, 17.4% had low, 67.5% had intermediate and 15.1% had high education; corresponding proportions for the angina sample were 14.5%, 67.6% and 17.9%. Participants with lower levels of education were older, more likely to live outside major cities, had more functional limitations and comorbidities and were less likely to have private health insurance.

**Table 1:** Sample characteristics by education level for patients admitted to hospital with acute myocardial infarction

	Education Level						Total	
	Low		Intermediate		High			
	%	n	%	n	%	n	%	n
Participants	17.4	774	67.5	3008	15.1	672	100	4454
Age, mean (SD)	75.4	11.3	73.3	11.7	70.3	11.2	73.2	11.6
45–54 years	4.3	33	6.1	183	7.7	52	6.0	268
55–64 years	16.2	125	21.2	639	28.1	189	21.4	953
65–74 years	25.2	195	26.3	790	30.8	207	26.8	1192
75–84 years	29.8	231	27.6	830	20.7	139	26.9	1200
≥85 years	24.6	190	18.8	566	12.7	85	18.9	841
Sex								
Male	53.9	417	63.9	1923	74.0	497	63.7	2837
Female	46.1	357	36.1	1085	26.0	175	36.3	1617
Remoteness								
Major cities	44.2	342	50.4	1515	56.9	382	50.3	2239
Inner regional	39.5	306	36.1	1087	31.9	214	36.1	1607
Outer regional/remote/very remote	15.0	116	11.7	353	9.2	62	11.9	531
Country of birth								
Australia/New Zealand	75.5	584	76.6	2305	73.4	493	75.9	3382
Other	23.0	178	22.1	665	26.2	176	22.9	1019
BMI								
Underweight	1.6	12	1.5	44	1.2	8	1.4	64
Normal weight	25.2	195	30.1	904	33.8	227	29.8	1326
Overweight	36.4	282	38.7	1163	41.5	279	38.7	1724
Obese	26.0	201	22.3	670	17.0	114	22.1	985
Physical functioning								
No or minor limitations	27.4	212	41.2	1240	59.8	402	41.6	1854
Moderate limitations	18.2	141	24.3	731	21.3	143	22.8	1015
Severe limitations	26.2	203	16.3	491	9.2	62	17.0	756
Comorbidities								
None	79.2	613	82.6	2485	89.7	603	83.1	3701
1	8.7	67	6.4	193	3.6	24	6.4	284
≥ 2	12.1	94	11.0	330	6.7	45	10.5	469
Private health insurance								
Yes	36.6	283	58.2	1751	77.4	520	57.3	2554
No	63.4	491	41.8	1257	22.6	152	42.7	1900

*n* Number of participants, *SD* Standard deviation

Note: Percentages (%) are given within each column/education group

Missing data: remoteness (1.7%); country of birth (1.2%); BMI (8%); physical functioning (18.6%); and comorbidities (10.5%)

Education attainment defined as low (no school certificate/qualifications), intermediate (school or leaving certificate/trade/apprenticeship/diploma/other certificate) and high (university degree or higher)

**Table 2:** Sample characteristics by education level for patients admitted to hospital with angina

	Education Level						Total	
	Low		Intermediate		High			
	%	n	%	n	%	n	%	n
Participants	14.5	631	67.6	2939	17.9	778	100	4348
Age, mean (SD)	71.6	10.4	70.5	10.2	68.4	10.1	70.3	10.3
45–54 years	6.2	39	6.6	194	8.2	64	6.8	297
55–64 years	22.2	140	25.1	738	30.2	235	25.6	1113
65–74 years	31.5	199	34.1	1003	37.0	288	34.3	1490
75–84 years	31.5	199	25.8	758	16.7	130	25.0	1087
≥85 years	8.6	54	8.4	246	7.8	61	8.3	361
Sex								
Male	48.3	305	55.4	1629	64.5	502	56.0	2436
Female	51.7	326	44.6	1310	35.5	276	44.0	1912
Remoteness								
Major cities	43.3	273	50.2	1476	61.3	477	51.2	2226
Inner regional	40.3	254	37.2	1093	29.7	231	36.3	1578
Outer regional/remote/very remote	15.1	95	11.2	328	7.2	56	11.0	479
Country of birth								
Australia/New Zealand	80.5	508	79.3	2330	71.5	556	78.1	3394
Other	18.5	117	19.6	577	27.5	214	20.9	908
BMI								
Underweight	0.6	4	1.0	29	0.8	6	0.9	39
Normal weight	20.4	129	25.8	758	29.4	229	25.7	1116
Overweight	38.4	242	40.3	1185	42.8	333	40.5	1760
Obese	27.9	176	25.1	738	21.2	165	24.8	1079
Physical functioning								
No or minor limitations	28.2	178	42.4	1245	58.5	455	43.2	1878
Moderate limitation	23.3	147	27.7	813	23.4	182	26.3	1142
Severe limitation	26.0	164	14.4	422	9.9	77	15.3	663
Comorbidities								
None	84.3	532	87.8	2580	90.7	706	87.8	3818
1	5.6	35	4.7	139	5.1	40	4.9	214
≥ 2	10.1	64	7.5	220	4.1	32	7.3	316
Private health insurance								
Yes	46.8	295	63.9	1879	84.1	654	65.0	2828
No	53.3	336	36.1	1060	15.9	124	35.0	1520

*n* Number of participants, *SD* Standard deviation

Note: Percentages (%) are given within each column/education group

Missing data: remoteness (1.5%); country of birth (1.1%); BMI (8.1%); physical functioning (15.3%); and comorbidities (7.3%)

Education level defined as low (no school certificate/qualifications), intermediate (school or leaving certificate/trade/apprenticeship/diploma/other certificate) and high (university degree or higher)

### Coronary procedures following admission with AMI

**Fig. 2:**
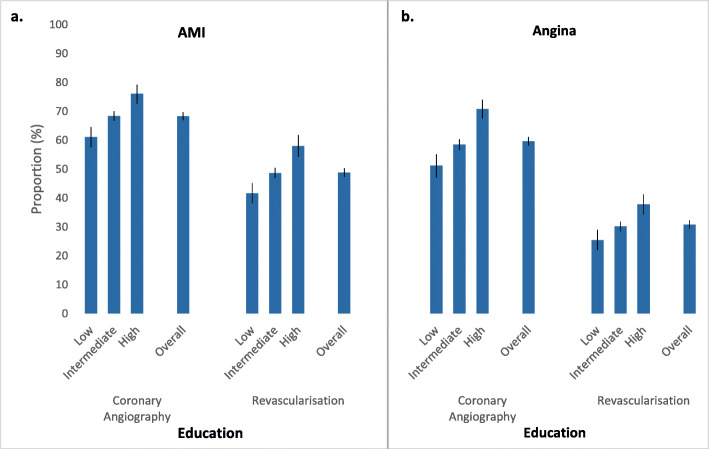
Proportion of (**a**) AMI and (**b**) angina patients who received coronary angiography or revascularisation by education level (low, intermediate or high). Proportions are given for all patients. Error bars depict the corresponding 95% confidence intervals. Education level defined as low (no school certificate/qualifications), intermediate (school or leaving certificate/trade/apprenticeship/diploma/other certificate) and high (university degree or higher). Revascularisation refers to percutaneous coronary intervention or coronary artery bypass grafting

Overall, 68.3% of AMI patients received angiography — low education = 61.1%, intermediate = 68.4%, high = 76.0%; 48.8% received a revascularisation procedure — low education = 41.6%, intermediate = 48.6%, high = 58.0% (Fig. 2a); and 5% died without receiving either procedure within 30 days of the index hospital admission. Among those who were still alive at the end of the 30-day follow-up period, 75.8 and 55.6% had received an angiogram or a revascularisation procedure, respectively. Following an AMI admission, crude rates per person-year were 25.8 for angiography and 11.7 for revascularisation procedures (Table 3). Crude rates of angiography ranged from 19.1 per person-year among those with low education to 37.4 per person-year for high education. Similar patterns were observed for revascularisation rates (Table 3).

**Table 3:** Coronary procedure rates and hazard ratios by education level (low, intermediate or high)

Procedure	Number of Procedures/py	Crude Procedure Rate per py (95% CI)	Adjusted HRs^**#**^ (95% CI)			
			Model 1	Model 2	Model 3	Model 4
***AMI patients (n = 4454)***						
*Coronary angiography*	3041/118	25.8 (24.9–26.7)				
Low	473/24.7	19.1 (17.5–21.0)	0.64 (0.53–0.78)	0.70 (0.57–0.85)	0.71 (0.56–0.90)	0.88 (0.71, 1.08)
Intermediate	2057/79.7	25.8 (24.7–27.0)	0.78 (0.67–0.91)	0.83 (0.71–0.97)	0.85 (0.71–1.02)	0.92 (0.79–1.09)
High	511/13.7	37.4 (34.3–40.8)	1	1	1	1
*p* (trend)			< 0.001	< 0.001	0.004	0.213
*Revascularisation*	2175/186.6	11.7 (11.2–12.2)				
Low	322/36.8	8.7 (7.8–9.8)	0.64 (0.53–0.78)	0.70 (0.57–0.85)	0.93 (0.69–1.25)	0.99 (0.73–1.34)
Intermediate	1463/126.4	11.6 (11.0–12.2)	0.75 (0.64–0.87)	0.78 (0.67–0.90)	0.89 (0.72–1.10)	0.91 (0.74–1.13)
High	390/23.5	16.6 (15.0–18.3)	1	1	1	1
*p* (trend)			< 0.001	< 0.001	0.514	0.821
***Angina patients (n = 4348)***						
*Coronary angiography*	2594/148.9	17.4 (16.8–18.1)				
Low	323/26.0	12.4 (11.2–13.9)	0.46 (0.40–0.55)	0.52 (0.44–0.62)	0.60 (0.47–0.76)	0.77 (0.60–0.98)
Intermediate	1720/103.2	16.7 (15.9–17.5)	0.59 (0.53–0.67)	0.65 (0.57–0.73)	0.70 (0.60–0.83)	0.79 (0.67–0.94)
High	551/19.8	27.9 (25.6–30.3)	1	1	1	1
*p* (trend)			< 0.001	< 0.001	< 0.001	0.0.16
*Revascularisation*	1340/250.8	5.3 (5.1–5.6)				
Low	160/38.6	4.1 (3.5–4.8)	0.63 (0.50–0.80)	0.71 (0.56–0.90)	0.78 (0.61–0.99)	0.79 (0.59–1.05)
Intermediate	886/170.9	5.2 (4.9–5.5)	0.77 (0.65–0.90)	0.84 (0.72, 1.00)	0.86 (0.73–1.02)	0.86 (0.71–1.04)
High	294/41.2	7.1 (6.4–8.0)	1	1	1	1
*p* (trend)			< 0.001	0.004	0.035	0.082

*CI* Confidence interval, *HR* Hazard ratio, *p p*-value, *py* Person-year

^#^**Model 1**: HRs age and sex-adjusted

**Model 2**: HRs adjusted for covariates in Model 1 + remoteness and country of birth

**Model 3**: HRs adjusted for covariates in Models 1 and 2 + BMI, physical functioning and comorbidities

**Model 4**: HRs adjusted for covariates in Models 1, 2 and 3 + private health insurance status

Education level defined as low (no school certificate/qualifications), intermediate (school or leaving certificate/trade/apprenticeship/diploma/other certificate) and high (university degree or higher)

Revascularisation refers to percutaneous coronary intervention or coronary artery bypass grafting

**Fig. 3:**
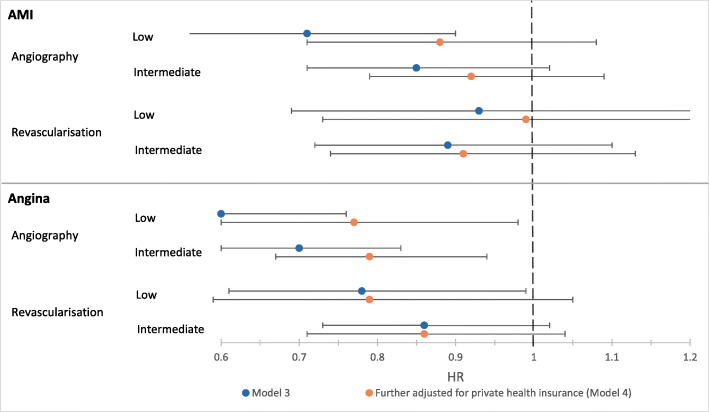
Forest plot showing hazard ratios for angiography and revascularisation by education level (low or intermediate; high = reference group), with (Model 4, [orange dot] and without (Model 3, [blue dot])) adjustment for private health insurance. Sample size = 4454 AMI patients; 4348 angina patients. Education level defined as low (no school certificate/qualifications), intermediate (school or leaving certificate/trade/apprenticeship/diploma/other certificate) and high (university degree or higher). Revascularisation refers to percutaneous coronary intervention or coronary artery bypass grafting. **Model 3**: HRs fully adjusted for age, sex, remoteness, country of birth, BMI, physical functioning and comorbidities. **Model 4**: HRs adjusted for covariates in Model 3 + private health insurance status. HR = hazard ratio; Error bars depict 95% confidence intervals

Rates of angiography were lower among those with lower levels of education, after adjusting for age and sex (Model 1) and remoteness and country of birth (Model 2), (linear test for trend *p* < 0.001; Table 3). Those with low compared to high education were 30% less likely to receive an angiogram (Model 2, HR = 0.70 [0.57–0.85]). Additional adjustment for health factors (BMI, physical functioning and comorbidities) made no material difference to the effect sizes. After additional adjustment for private health insurance, the HRs decreased and there was little evidence of an association between education and coronary procedures (Fig. 3).

Rates of revascularisation were also lower among those with lower levels of education after adjusting for demographic factors (Table 3), however, this association was no longer evident after adjustment for health factors (Model 3, low vs high HR = 0.93 [0.69–1.25]), and adjusting for private health insurance made little difference (Fig. 3). For the association between education and revascularisation among AMI patients, HRs were further from the null in the earlier time period (2006–10; HR for low vs. high education = 0.66 [0.26–1.73]) compared to the later time period (2011–16; HR low vs. high education = 0.80 [0.52–1.22]), but results were not significant. Results were similar when restricted to the subset of AMI patients who had received angiography (Additional file 2).

### Coronary procedures following admission with angina

Overall, 59.7% of patients received a coronary angiogram, 30.8% received revascularisation and 0.2% died without receiving either procedure within 30 days of hospitalisation for angina (Fig. 2b). Proportions receiving angiography and revascularisation were lower among those with lower levels of education — angiography: 51.2 and 58.5% vs. 70.8% for low and intermediate vs. high education; revascularisation: 25.4 and 30.2% vs. 37.8% for low and intermediate vs. high education. Following admission with angina, the overall crude rate for angiography per person-year was 17.4 and ranged from 12.4 for patients with low education to 27.9 for those with high education (Table 3). Crude rates for revascularisation procedures were 5.3 per person-year overall and ranged from 4.1 per person-year for patients with low education to 7.1 per person-year for patients with high education.

After adjusting for age and sex (Model 1) and remoteness and country of birth (Model 2), HRs for angiography and revascularisation procedures were lower for patients with lower levels of education (tests for trend: *p* < 0.001; Table 3)—compared to those with high education, angiography rates were 48% lower (Model 2, 0.52 [0.44–0.62]) and revascularisation 29% lower (Model 2, 0.71 [0.56–0.90]) among patients with low education. HRs attenuated slightly after adjustment for BMI, physical functioning and comorbidities (Model 3). Additional adjustment for private health insurance (Model 4) further reduced the magnitude of the HRs (Table 3). There was no evidence of education-related variation in revascularisation procedures among angina patients who received angiography (Additional file 2).

## Discussion

In this large-scale Australian prospective cohort study, there was a socioeconomic gradient in coronary procedure rates following hospital admission for AMI or angina. The likelihood of receiving a procedure decreased with increasing disadvantage, such that those with the lowest compared to the highest education were around 30% less likely to receive an angiogram, and 30% less likely to receive a revascularisation procedure if admitted with AMI, and 50% less likely if admitted with angina. Broad consideration of health (BMI, physical functioning and hospital-recorded comorbidities) did not explain these differences, except for the differences in revascularisation rates among AMI patients. Private health insurance accounted for some but not all of the education-related variation in procedure rates. Our study examined socioeconomic differences in procedure rates averaged over the study period. Although we did not find evidence of inequality in revascularisation among AMI patients, inequalities may have decreased over time, in line with diffusion of innovation theory [16]. However, our study did not aim to look at changes in inequalities over time and was insufficiently powered to do this.

Previous Australian studies have reported mixed findings regarding socioeconomic variation in the receipt of angiography, ranging from no evidence of an association [16, 21], to statistically non-significant findings in the same direction as those observed in our study [22]. These studies used area- rather than individual-level data and were restricted to public hospital data only, which may have underestimated socioeconomic variation in procedure rates. Our findings are similar to those reported in international studies using area- and individual-level measures, which have found that low socioeconomic position is associated with delays in receiving angiography [36] and lower angiography procedure rates [20, 37, 38]. Our finding that socioeconomic differences in revascularisation rates among those with angina were substantially attenuated after accounting for angiography is also consistent with previous studies. A Norwegian study (2001–2009) found that socioeconomic variation in revascularisation rates could be fully explained by differences in the receipt of angiography [20]. Socioeconomic differences are most apparent for diagnostic procedures and tend to diminish with subsequent IHD treatment such as revascularisation [10]. Taken together, these findings suggest that, among angina patients where coronary procedures are less protocol-driven than for AMI patients [39], socioeconomic variation is more evident during diagnostic stages (angiogram) but, once investigated, patients receive the same level of revascularisation regardless of their socioeconomic position.

Private health insurance is one possible mechanism through which socioeconomic inequalities in provision of care may arise in Australia. In our study, people with higher education were more likely to hold private health insurance than those with less education, and consistent with earlier Australian studies, [15, 40] privately insured patients were more likely to receive angiography. Remuneration for angiography to health care providers within the private health sector in Australia is fee-for-service and therefore is based on the quantity, not quality, of care provided [40], and the ratio of angiography to revascularisation is greater within the private health care system compared to the public health sector [41]. This suggests possible over-utilisation among privately insured patients, but it may reflect under-utilisation among public patients. This relative over- or under-utilisation could also reflect differing patient and clinician expectations [42–45].

The observed socioeconomic gradient in procedure rates may also reflect differences in clinical need for angiography. Current Australian guidelines do not recommend angiography for all AMI and angina patients, rather, indication depends on disease severity, risk of future events and other clinical characteristics. For example, patients with non-ST-elevation MI are indicated for angiography only if they are at immediate or high risk of a future cardiovascular event [11] and it is generally indicated in patients with stable angina only when symptoms cannot be managed with medication [46, 47]. A number of clinical characteristics, such as the presence of major comorbidities, are contraindications for angiography. Although we adjusted for related factors, we cannot exclude the possibility that the low education group within our study sample comprised a greater proportion of patients in whom angiography was not indicated.

The use of individual-level data on education to measure patients’ socioeconomic position was a major strength of this study. Together with the inclusion of data from both public and private hospitals, and information on private health insurance, this enabled possibly more accurate estimates of socioeconomic differences in coronary procedure rates compared to previous Australian studies. Linkage of questionnaire data to hospital administrative and death records allowed for virtually complete follow-up of patients over time and provided more detailed information on relevant variables and confounders of interest than administrative data alone. However, data on certain clinical characteristics of the patients were not available, including the disease severity at time of admission and risk of a future cardiovascular event. Data did not allow distinction between subtypes of AMI and angina. As such, it was not possible to assess whether care reflected guideline recommendations. We adjusted for comorbidities, BMI and physical functioning in an attempt to account for contraindications to coronary procedures, but a degree of residual confounding is likely. Although education is just one measure of socioeconomic position, it is a reliable measure at the individual-level and is relatively stable over time, although the relative value of education varies across age cohorts. Finally, procedure rates are not necessarily generalisable because the study cohort is not wholly representative of the general NSW population, however relative measures of association from cohort studies are broadly applicable to the general population [27, 48].

## Conclusions

Socioeconomic variation in the provision of health care can signal potential points of intervention to improve outcomes for disadvantaged groups. Using linked data from public and private hospitals and an individual-level measure of education, this study has shown that there is a socioeconomic gradient in the receipt of angiography following an AMI or angina admission, with the most disadvantaged people less likely to receive an angiogram compared to those with the least disadvantage. Some of this variation was explained by differences in private health insurance between socioeconomic groups. Other reasons for lower rates of angiography, including the extent to which the variation is clinically appropriate or reflects under treatment of those from low socioeconomic position or over treatment of those from a high socioeconomic position, remain unclear.

## Supplementary information

**Additional file 1.** ACHI procedure codes used to identify receipt of coronary procedures. Table containing the procedure codes used to identify receipt of coronary angiography and revascularisation from linked hospitalisation records.

**Additional file 2.** Revascularisation rates and hazard ratios by education level (low, intermediate or high), restricted to patients who received an angiogram. Supplementary table displaying the model results of the relationship of education to revascularisation, restricted to those who received an angiogram.

## Data Availability

Access to the Sax Institute’s 45 and Up Study data is available to any bona fide researcher who has a scientifically sound and feasible research proposal; has ethics approval for the proposal and data custodian approval for access to linked data, if required for the project; and can meet 45 and Up Study licence and SURE (Secure Unified Research Environment) user charges. See https://www.saxinstitute.org.au/our-work/45-up-study/for-researchers.

## Abbreviations

ACHI: Australian Classification of Health Interventions
AMI: Acute myocardial infarction
APDC: NSW Admitted Patient Data Collection
ARIA: Accessibility/Remoteness Index of Australia
BMI: Body mass index
CABG: Coronary artery bypass grafting
CI: Confidence interval
CVD: cardiovascular disease
HR: Hazard ratio
ICD-10-AM: International Statistical Classification of Diseases and Related Health Problems, Tenth Revision, Australian Modification
IHD: Ischaemic heart disease
n: Number of participants
NSW: New South Wales
p: *p*-value
PCI: Percutaneous coronary intervention
PF-10: Medical Outcomes Study Physical Functioning Subscale
py: Person-year
SD: Standard deviation
