# Supplementary material for: Education-related variation in coronary procedure rates and the contribution of private health care in Australia: a prospective cohort study
Source: Int J Equity Health. 2020 Aug 14;19:139. doi: 10.1186/s12939-020-01235-y (PMC7427777; doi:10.1186/s12939-020-01235-y)
Supplement: Supplementary file 1 — Additional file 1. ACHI procedure codes used to identify receipt of coronary procedures. Table containing the procedure codes used to identify receipt of coronary angiography and revascularisation from linked hospitalisation records. [file 12939_2020_1235_MOESM1_ESM.docx]

**Additional file 1.** ACHI procedure codes used to identify receipt of coronary procedures.

| **Coronary Procedure** | **ACHI Code** |
| --- | --- |
| Coronary angiography | 38215, 38218 |
| PCI | 35304-00, 35305-00, 35304-01, 35305-01, *38300-00, 38303-00* (block: 670);  35310-00, 35310-01, 35310-02, 35310-03, 35310-05, *38306-00, 38306-01, 38306-02, 38306-03, 38306-05* (block: 671) |
| CABG | 38497-00 to 38497-07, 38500-00 to 38500-04, 38503-00 to 38503-04, 90201-00 to 90201-03, *38500-05, 38503-05* (blocks 672-679) |

ACHI = Australian Classification of Health Interventions (8^th^ edition); PCI = percutaneous coronary intervention; CABG = coronary artery bypass grafting

Procedures identified from the 50 procedure code fields in linked hospitalisation records.
