# Supplementary material for: Education-related variation in coronary procedure rates and the contribution of private health care in Australia: a prospective cohort study
Source: Int J Equity Health. 2020 Aug 14;19:139. doi: 10.1186/s12939-020-01235-y (PMC7427777; doi:10.1186/s12939-020-01235-y)
Supplement: Supplementary file 2 — Additional file 2. Revascularisation rates and hazard ratios by education level (low, intermediate or high), restricted to patients who received an angiogram. Supplementary table displaying the model results of the relationship of education to revascularisation, restricted to those who received an angiogram. [file 12939_2020_1235_MOESM2_ESM.docx]

**Additional file 2.** Revascularisation rates and hazard ratios by education level (low, intermediate or high), restricted to patients who received an angiogram.

| **Sample** | **Number of Procedures/py** |  | **Crude Procedure Rate per py (95% CI)** |  | **Adjusted HRs^#^ (95% CI)** | | | | | | |
| --- | --- | --- | --- | --- | --- | --- | --- | --- | --- | --- | --- |
|  |  |  |  |  | **Model 1** |  | **Model 2** |  | **Model 3** |  | **Model 4** |
| *AMI* | 2106/91.0 |  | 23.1 (22.2-24.1) |  |  |  |  |  |  |  |  |
| Low | 306/16.2 |  | 18.9 (16.9-21.2) |  | 0.74 (0.59–0.94) |  | 0.76 (0.60–0.97) |  | 0.83 (0.65–1.07) |  | 0.83 (0.64–1.06) |
| Intermediate | 1416/62.1 |  | 22.8 (21.6-24.0) |  | 0.77 (0.64–0.92) |  | 0.78 (0.65–0.94) |  | 0.82 (0.68–0.99) |  | 0.82 (0.68–0.99) |
| High | 384/12.7 |  | 30.1 (27.3-33.3) |  | 1 |  | 1 |  | 1 |  | 1 |
| *p* (trend) |  |  |  |  | 0.013 |  | 0.023 |  | 0.128 |  | 0.114 |
| *Angina* | 1226/123.4 |  | 9.9 (9.4-10.5) |  |  |  |  |  |  |  |  |
| Low | 140/16.0 |  | 8.8 (7.4-10.3) |  | 0.93 (0.72-1.20) |  | 0.95 (0.73-1.23) |  | 0.97 (0.74-1.26) |  | 0.90 (0.69-1.18) |
| Intermediate | 810/82.1 |  | 9.9 (9.2-10.6) |  | 0.95 (0.80-1.12) |  | 0.98 (0.82-1.16) |  | 0.97 (0.81-1.15) |  | 0.93 (0.78-1.11) |
| High | 276/25.3 |  | 10.9 (9.7-12.3) |  | 1 |  | 1 |  | 1 |  | 1 |
| *p* (trend) |  |  |  |  | 0.507 |  | 0.694 |  | 0.751 |  | 0.393 |

CI = confidence interval; HR = hazard ratio; n= number of participants; *p* = p-value; py = person-year

^#^**Model 1**: HRs age and sex-adjusted

**Model 2**: HRs adjusted for covariates in Model 1 + remoteness and country of birth

**Model 3**: HRs adjusted for covariates in Models 1 and 2 + BMI, physical functioning and comorbidities

**Model 4**: HRs adjusted for covariates in Models 1, 2 and 3 + private health insurance status

Education level defined as low (no school certificate/qualifications), intermediate (school or leaving certificate/trade/apprenticeship/diploma/other certificate) and high (university degree or higher).

Revascularisation refers to percutaneous coronary intervention or coronary artery bypass grafting.
